# The biodistribution of placental and fetal extracellular vesicles during pregnancy following placentation

**DOI:** 10.1042/CS20220301

**Published:** 2023-03-15

**Authors:** Matthew Kang, Cherie Blenkiron, Lawrence W. Chamley

**Affiliations:** 1 Department of Obstetrics and Gynaecology, University of Auckland, 1023, Auckland, New Zealand; 2 Hub for Extracellular Vesicle Investigations (HEVI), University of Auckland, 1023, Auckland, New Zealand; 3 Auckland Cancer Society Research Center (ACSRC), University of Auckland, 1023, Auckland, New Zealand; 4 Molecular Medicine and Pathology, University of Auckland, 1023, Auckland, New Zealand

**Keywords:** biodistribution, exosome, extracellular vesicle, microparticles, placenta, pregnancy

## Abstract

Human pregnancy is a highly orchestrated process requiring extensive cross-talk between the mother and the fetus. Extracellular vesicles released by the fetal tissue, particularly the placenta, are recognized as important mediators of this process. More recently, the importance of placental extracellular vesicle biodistribution studies in animal models has received increasing attention as identifying the organs to which extracellular vesicles are targeted to helps us understand more about this communication system. Placental extracellular vesicles are categorized based on their size into macro-, large-, and small-extracellular vesicles, and their biodistribution is dependent on the extracellular vesicle’s particle size, the direction of blood flow, the recirculation of blood, as well as the retention capacity in organs. Macro-extracellular vesicles are exclusively localized to the lungs, while large- and small-extracellular vesicles show high levels of distribution to the lungs and liver, while there is inconsistency in the reporting of distribution to the spleen and kidneys. This inconsistency may be due to the differences in the methodologies employed between studies and their limitations. Future studies should incorporate analysis of placental extracellular vesicle biodistribution at the macroscopic level on whole animals and organs/tissues, as well as the microscopic cellular level.

## Introduction

Human pregnancy is characterized by systemic alterations to the cardiovascular system, including a 30–50% increase in cardiac output and reduced peripheral vascular resistance, and immunomodulation to facilitate the tolerance of the semi-allogenic fetus [1]. These processes are highly orchestrated requiring cross-talk between the mother and the fetus which can occur directly at the physical fetal–maternal interface that is the placental bed where the placenta implants into the uterus, but also systemically through the release of soluble factors, including cytokines, chemokines, and hormones [2].

In more recent years, however, growing attention has focused on the role of extracellular vesicles as mediators of intercellular communication during pregnancy. Extracellular vesicles (EVs) are a heterogeneous collection of phospholipid-enclosed particles that carry a diverse range of cargo including lipids, proteins, RNAs and DNA. EVs are released from all cells studied to date, including the trophoblasts of the placenta and the embryo, both of which are fetal tissues [3]. EVs have historically been classified into three populations based on their size and routes of biogenesis – apoptotic bodies (∼1 µm) produced during apoptotic cell disassembly, microvesicles (150–1000 nm) generated through the blebbing of the plasma membrane and exosomes (<150 nm) created in multivesicular bodies of the late endosomal pathway [4]. However, due to the challenges in confirming the exact origin of EVs within cells and the now appreciated overlap in sizes between these traditional subtypes, it is now recommended to use terms based on their size alone – large-EVs for EVs between 150 and 1000 nm and small-EV for those up to 150 nm [5]. The placental syncytiotrophoblast also release extremely large particles that are approximately 70 µm in diameter, termed macro-EVs [6]. These terms will be applied in this review.

Given that human pregnancy is characterized by a substantial increase in total small-EV concentration in the maternal circulation (∼13-fold in women of 28 weeks gestation) compared with non-pregnant women [7], it is no surprise that there has been a large number of studies investigating the potential roles of pregnancy-related EVs, although typically these studies are *in vitro*. The known functions of placental EVs were succinctly reviewed in Tannetta et al. [3]. However, *in vitro* EV studies often use unnaturally high EV concentrations which are added to cells in a static culture that may cause a non-physiologic interaction with recipient cells [8]. These studies are typically performed on one cell type at a time without consideration of cells that would be in close anatomic proximity *in vivo*. As such, *in vitro* reports can be exaggerated and describe interactions of EVs and cells that are out of context. The physiological context of EV function requires the knowledge of their intended target cell(s), which is ultimately, the purpose of EV biodistribution studies. The study of the biodistribution of placental EVs has gained significant traction in the last decade. This review summarizes the current knowledge on the bi-directional EV biodistribution between the mother and the fetus during pregnancy after implantation. Studies that have investigated the biodistribution of placental or fetal EVs in the maternal and/or fetal compartments and the techniques used to identify biodistribution are listed in Table 1. There is also good evidence that there is cross-talk between the preimplantation embryo and the uterus but that topic is reviewed elsewhere [9].

**Table 1:** Studies that have investigated the biodistribution of pregnancy-related extracellular vesicles in animal models with and without pregnancy

Reference	EV type	Donor source	Recipient animal	Route of administration (dose or concentration)	Timepoints investigated	Analysis method	Organs analyzed	Main findings
[83]	Small-EVs	**Uterine flush-derived EVs:** Day 14 cyclic sheep uterine flush	**Uterine flush-derived EVs:** Sheep (day 8 of post-mate)	**Uterine flush-derived EVs:** Infusion by osmotic pump implanted at the uterine horn (5.6 × 10^10^ particles over 6 days)	**Uterine flush-derived EVs:** 6 days (day 14 of post-mate)	Epifluorescent microscopy of tissue sections	**Uterine flush-derived EVs:** Uterus, ovaries, parametrial lymph nodes, liver, lung	**Uterine flush-derived EVs:** Localized to the conceptus (trophectoderm), uterine epithelium, but not the uterine stroma or myometrium, ovary, corpus luteum, parametrial lymph node, lung
		**Conceptus-derived EVs:** Day 14 conceptus	**Conceptus-derived EVs:** Sheep (day 8 of post-estrus)	**Conceptus-derived EVs:** Infusion by osmotic pump implanted at the uterine horn (5.8×10^10^ particles over 6 days)	**Conceptus-derived EVs:** 6 days (day 14 of post-estrus)		**Conceptus-derived EVs:** Uterus, ovaries, parametrial lymph nodes, liver, lung	**Conceptus-derived EVs:** Localized to the uterine epithelia, but not in uterine stroma or myometrium, ovary, corpus luteum, parametrial lymph node, lung
[15]	Small-EVs	Human amnion epithelial cell	Pregnant CD1 mice (gestation day 17)	Amniotic cavity (random)	24 h (gestation day 18)	Bruker In Vivo MS FX PRO Imager IVIS imager	Placenta, uterus, kidneys, serum	Localized to the maternal side of the placenta, uterus, serum, kidneys
[57]	Small-EVs	Human first-trimester placental explant	Pregnant CD1 mice (gestation day 12.5)	Tail vein (100 µg total protein weight)	30 min, 24 hrs	IVIS imager	Liver, lungs, kidneys, spleen, brain, thymus, heart, pancreas, muscle	**After 30 min:** Localize to the liver and lungs **After 24 h:** Localize to the liver, lungs, kidneys
[84]	Small-EVs	Circulating small-EVs isolated from the serum of normal mice	Pregnant C57BL/6J (gestation day 14.5 and 16.5)	Tail vein (40 µg total protein weight)	Not specified	Confocal laser scanning microscopy of tissue sections	Fetal heart, placenta	**At both gestation day 14.5 and 16.5:** Sparsely localized to the placenta and fetal heart
[85]	Small-EVs	Circulating small-EVs isolated from the plasma of pregnant CD1 mice (gestation day 9 and 18)	Pregnant CD1 mice (gestation day 15)	i.p. (three doses of 3.33 × 10^10^ or 9.16 × 10^10^ particles over 24 hours)	∼48 h (gestation day 17)	Olympus BX43 fluorescent microscopy of tissue sections	Cervix, uterus, fetal membranes, placenta	**For both gestation day 9 and 18 small-EVs:** Localized to the cervix, uterus, fetal membranes, placenta
[16]	Small-EVs	Pregnant transgenic C57BL/6J mouse carrying fetus/fetal tissue expressing tomato RFP and EGFP construct	Identical to the donor animal	Natural passage of EVs from conceptus to the maternal circulation	16 days (gestation day 16)	Bead-coupled flow cytometry for analysis of maternal plasma Confocal microscopy for analysis of maternal tissue	Maternal plasma Maternal uterus, cervix	Fetal small-EVs constituted 35% (5.66 × 10^9^ particles) of total small-EVs (1.62 × 10^10^) in the maternal circulation Fetal small-EVs localized to the uterus and cervix
	Small-EVs (Cre-enriched)	HEK293T cells transfected with Cre-mcherry-CRY2 vector	Pregnant transgenic C57BL/6J mouse (as above) (gestation day 13)	i.p. (1 × 10^10^ particles)	72 h (gestation day 16)	Confocal microscopy	Placenta, fetal membrane	Small-EVs localized to the placenta and fetal membrane
[58]	Small-EVs	Non-pregnant C57BL/6J mice plasma	Non-pregnant C57BL/6J mice	Tail vein (2.5 × 10^10^ particles)	30 min	Epifluorescent microscopy of tissue sections	Lungs, liver	**EVs from non-pregnant mice plasma:** Readily detectable in the liver, but not detectable in lungs
		Pregnant C57BL/6J mice plasma (gestation day 14.5)				t-SNE flow cytometry		**EVs from pregnant mice plasma:** Readily detectable in the liver and lungs
		C57BL/6J mice placental explant (gestation day 14.5)	Non-pregnant C57BL/6J mice	Tail vein (2.5 × 10^10^ particles)	30 min	Immunofluorescence microscopy of tissue sections	Lungs, liver	**Using both techniques:** EVs localized predominantly in the lung interstitial macrophages but not alveolar macrophages, and in the liver endothelial cells and Kupffer cells
		C57BL/6J mice placental explant (gestation day 14.5)	Non-pregnant C57BL/6J mice	Tail vein (2.5 × 10^10^ particles)	24 h	LiCor whole organ imager	Lung, brain, thymus, heart, kidney, para-aortic lymph node, spleen, small intestine, uterus, ovaries	EVs localized to the lungs, but not in any other examined tissues
		Pregnant transgenic C57BL/6J mouse carrying female pups and their placenta (Cre-positive) that ubiquitously express GFP	Identical to the donor animal	Natural passage of EVs from conceptus to the maternal circulation	14.5 days (gestation day 14.5)	Confocal microscopy of tissue sections	Lungs	Fetal EVs readily localized to the lung
[40]	Large-EVs and Small-EVs	Human first-trimester placental explant (control IgG-treated) Human first-trimester placental explant (antiphospholipid antibody-treated) Human term placental explant (normotensive) Human term placental explant (antiphospholipid antibody-treated)	Pregnant CD1 mice (gestation day 12.5–13.5)	Tail vein	30 min	AMI HTX spectral imager	Ovary, fetus, placenta, mesentery, liver, hepatic lymph node, spleen, kidneys, renal lymph node, thymus, heart, lungs, skeletal muscle, brain, urine	**First-trimester large- and small-EVs regardless of treatment by antiphospholipid antibody:** Primarily to the liver, followed by the lungs, kidneys, and spleen **Term large- and small-EVs regardless of treatment by antiphospholipid antibody:** Same as above
[34]	Large-EVs	Human first-trimester placental explant	Non-pregnant CD1 mice	**Tail vein (In non-pregnant mice:** 300 µg total protein weight	2 min, 30 mins, 24 h	IVIS imager	Brain, thymus, heart, lungs, liver, spleen, pancreas, kidneys, uterus/placenta, skeletal muscle	**In non-pregnant mice, large-EVs localized to:** **After 2 min: lungs only** **After 30 min:** lungs, liver, kidneys **After 24 h:** liver, kidneys
			Pregnant CD1 mice (gestation day 12.5)	**In pregnant mice:** 100 µg				**In pregnant mice, large-EVs localized to:** **After 30 min:** lungs only **After 24 h:** lungs, liver
								**In pregnant mice, macro-EVs localized to:** At all time points: exclusively to the lungs
[29]	Necrotic cell suspension of JEG-3 cells	Necrotic JEG-3 cells	Non-pregnant female Wistar rats	Femoral vein (∼2.5 × 10^6^ cells) Jugular vein (∼2.5 × 10^6^ cells)	5 min	IVIS imager	Thymus, heart, lungs, kidney, liver, reproductive organs	**Both routes of injection:** trophoblastic debris localized to the lungs only

Abbreviations: Cre, Cre recombinase; EGFP, enhanced green fluorescent protein; EV, extracellular vesicle; IgG, immunoglobulin G antibody; i.p. intraperitoneal; RFP, red fluorescent protein.

## Placenta-derived EVs are the major fetal EV population in the maternal circulation during pregnancy

The fetal-maternal cross-talk during pregnancy likely involves a complex web of interactions between numerous cell types of maternal and fetal origins. The syncytiotrophoblast is a multinucleated cell that (a) covers the entire maternal-facing surface of the placenta (surface area of 11–13 m^2^ at term), (b) is bathed in maternal blood throughout most of gestation, and (c) releases a diverse array of cellular particles (previously coined ‘trophoblast debris’) directly into the maternal blood. These particles include multinucleated syncytial nuclear aggregates (SNAs), mononuclear trophoblasts, apoptotic bodies, trophoblast ghosts, and other smaller EVs [10–12]. Thus, the syncytiotrophoblast is a major source of placenta-derived EVs [13,14]. In addition to the syncytiotrophoblast, villous cytotrophoblasts are also at least temporarily in contact with the maternal blood at sites of syncytial denudation and endovascular trophoblasts in the maternal spiral arteries are minor sources of placental EVs [10]. Although the embryo or fetus can also contribute EVs that pass through the placental barrier into the maternal circulation [15,16], most of the fetal-derived EVs in the maternal circulation are derived from placental trophoblasts. Placental EVs can be seen in the maternal circulation, identified by the placental-type alkaline phosphate (PLAP) marker, from as early as 6 weeks of gestation [17], with numbers increasing throughout pregnancy [18–20].

**Figure 1:**
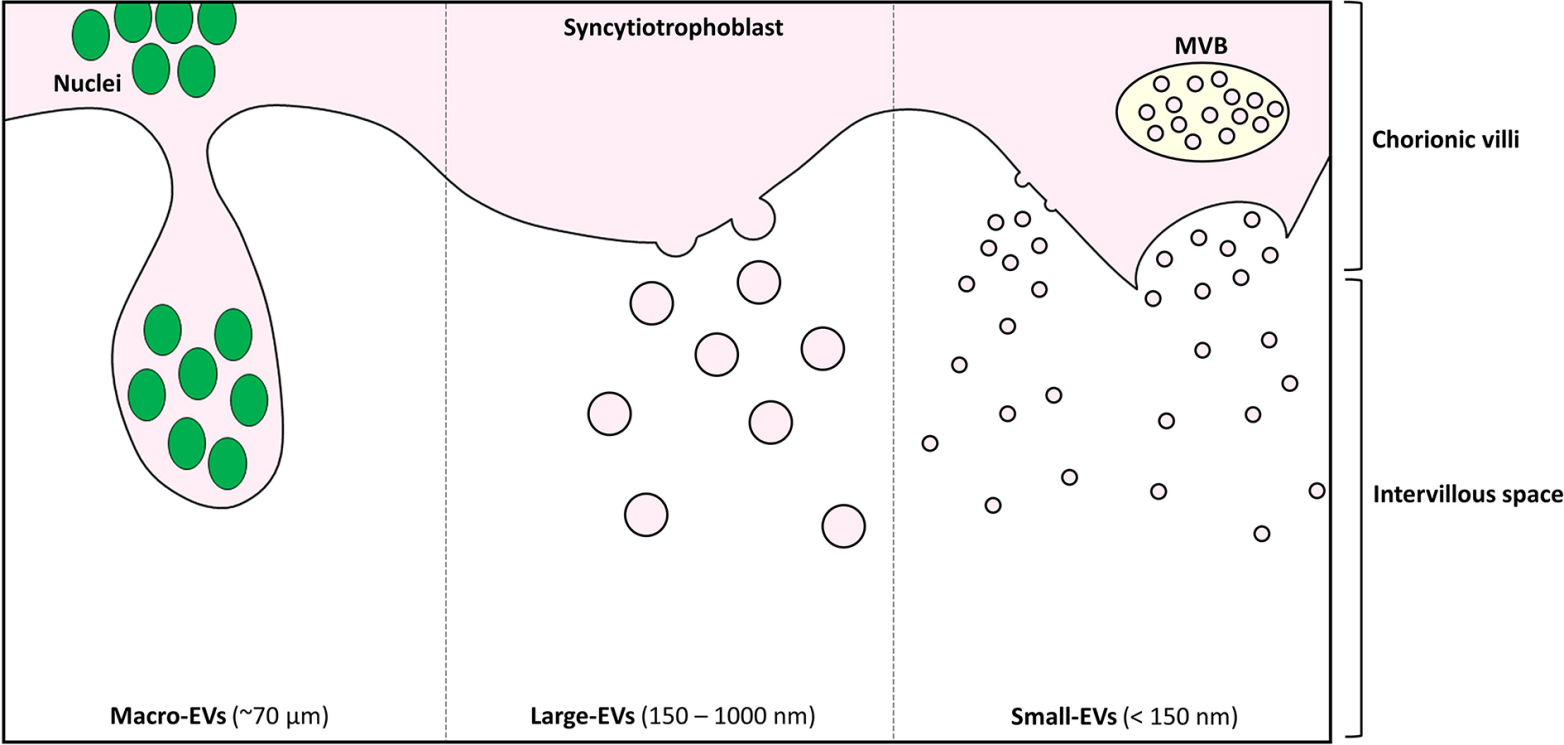
Schematic representation of the different types of extracellular vesicles shed from the placental syncytiotrophoblast into the maternal circulation. Macro-EVs include the multinucleated SNAs. Large-EVs are largely generated at the plasma membrane surface through blebbing, while small-EVs are generated through both plasma membrane surface blebbing and through multivesicular bodies (MVB) of the late endosomal pathway.

To simplify the apparent diversity of EVs released by the placenta, EVs can be categorized into three broad types based on their size: (a) macro-EVs (which includes SNAs), (b) large-EVs, and (c) small-EVs (which includes exosomes). These EV types are illustrated in Figure 1.

### Placental macro-EVs

In 1893, Schmorl first reported that large multinucleated structures which he referred to as *plazentazellen* were trapped in the small pulmonary blood vessels of pregnant women [6,21]. Today these structures are referred to as SNAs or macrovesicles. These structures are frequently teardrop in shape and have an average diameter of 70 µm [6]. Thus, to our knowledge SNAs are the largest of the known EVs in the literature which we call macrovesicles (macro-EVs). While macro-EVs are released from the placenta in all pregnancies there is a 20-fold increase in their number in pregnancies complicated by preeclampsia [22,23]. Although the exact biogenesis of macro-EVs is unclear, two mechanisms have been proposed. First, macro-EVs may result from the detachment of newly forming placental villi from the placental surface [10]. Second, macro-EVs may represent the end stage of a programmed cell death process in the multinucleated syncytiotrophoblast [10,24]. In this scenario, macro-EVs would be functionally equivalent to apoptotic bodies produced by mononuclear cells in the terminal phase of apoptotic cell death. It is possible that macro-EVs in the maternal blood/lungs are derived from both mechanisms. The production of macro-EVs is essentially unique to higher primate pregnancy with few other animals having been reported to produce similar structures from their placentae.

### Placental large- and small-EVs

Although the exact mechanisms underlying the biogenesis of placental large-EVs is unknown, morphological observations by electron microscopy indicate that they are constitutively released from the apical side of the syncytiotrophoblast at the microvilli [25,26]. Other reports have indicated that one-third of SNAs themselves release large-EV sized vesicles, although this would be a very minor source of large-EVs [12,27].

Placental small-EVs encompass exosomes that originate from the late endosomal system [4], but also nano-sized EVs that originate through other routes of biogenesis, e.g. through blebbing at the surface membrane of the syncytiotrophoblast. The concentrations of both large- and small-EVs collected from placental explants increase substantially (∼100-fold) from 8 to 12 weeks of gestation [18], while up to ∼8-fold increase in concentrations have been reported in the maternal blood from first-trimester to third-trimester [19,20].

## The biodistribution of placental macro-EVs in women and animal models

Macro-EVs were first reported to be localized in the lungs of women who had died during pregnancy approximately 120 years ago [6]. Macro-EVs travel from the placenta via large veins until they become physically lodged in the first small vessels they encounter in the maternal lungs. A comparison of the numbers of macro-EVs in the uterine and peripheral blood confirmed that vast majority of these very large EVs do not pass through the maternal lungs into the periphery [28] and substantial numbers of them have only been reported in human maternal lungs and no other organs [29,30]. Quantification of macro-EVs in maternal lungs pre- and post-partum confirmed that macro-EVs are rapidly cleared, usually in 3–4 days [30]. One estimate suggests that approximately 100,000 macro-EVs are extruded from the normal placenta each day [31] with another estimate suggesting that at 12 weeks of gestation there are approximately 50,000 macro-EVs released into the maternal blood daily rising to 800,000 at term [32]. The number of macro-EVs extruded from the placenta is reported to increase 20-fold in women with preeclampsia [33]. Placental macro-EVs are particularly important for understanding the biodistribution of placental EVs as, to our knowledge, these are the only EVs whose biodistribution has been characterized in women. This means that placental macro-EVs can be used as an important positive control to demonstrate that animal models accurately reflect that biodistribution of EVs in humans. This was the case for Tong et al., who injected human macro-EVs into pregnant and non-pregnant mice and found that these vesicles were localized exclusively to the lungs [34].

Although the exact physiological processes responsible for clearing macro-EVs from the pulmonary vessels requires further investigation, pulmonary endothelial cells may participate through phagocytosing these EVs while macro-EVs may also undergo further blebbing into smaller particles which may effectively reduce the macro-EV size [27,35,36]. It was previously suggested that there may be a syncytiolysin that dissolves the macro-EV in the lungs but there is little or no evidence supporting this hypothesis [30].

The physiological role of macro-EVs requires further elucidation, especially in the context of the maternal lung. In a normal healthy pregnancy, macro-EVs are cleared from the maternal circulation without generating an inflammatory response, and there is evidence macro-EVs carry markers of programmed cell death such that they may be involved in mediating anti-inflammatory or tolerogenic responses to fetal antigens [37] as is the case for apoptotic bodies from other cells [38]. Macro-EVs may also contribute to the pathology of pregnancy complications such as preeclampsia, via the transfer of aberrant EV cargos, including ‘danger signals/alarmins’ and miRNAs that can dysregulate gene expression in recipient endothelial cells and lead to their activation *in vitro* [35,39].

## The biodistribution of placental large-EVs

To date, the two published studies that have investigated the biodistribution of placental large-EVs have utilised EVs derived from human placental explant culture in mouse models (Table 1). These studies have led to the following findings.

### Placental large-EVs localize to the lungs and the liver

The lungs and the liver are the major sites in which placental large-EVs were detected. Following tail vein administration into non-pregnant and pregnant mice, human placental large-EVs are seen exclusively in the lungs at 2 min, with gradually decreasing levels in the lungs until 24 h with concomitantly increasing detection in the liver and to a lesser extent other organs [34,40]. This may not be a placenta-specific pattern of EV biodistribution as intravenous (i.v.) administration of both small- and large-EVs from other sources including 4T1 cells, macrophages, and dendritic cells were also reported to result in exclusive localization in the lungs very early on (3 min) following tail vein administration [41], and moderate levels of detection in the lungs and concomitant high levels of detection in the liver of non-pregnant mice at ∼4 h [42,43]. This time-dependent change in biodistribution probably reflects the initial entrapment of EVs in the lungs as the organ with the first capillary bed in which injected EVs must pass through before disseminating to the liver and other organs. While the localization of large-EVs to the maternal lungs is in part likely due to the first-pass effect, it is equally clear that despite vast quantities of blood passing through the lungs, significant amounts of placental EVs are retained in the lungs suggesting a specific interaction of the EVs with pulmonary cells. As the resting mouse passes its entire blood volume around the body 7–8 times/minute [44], that the movement of some large-EVs from the lungs is delayed for up to 24 h also suggests that there is a transient interaction between the EVs and pulmonary cells.

During pregnancy, the maternal lungs undergo anatomical and functional changes, including changes to the extracellular matrix (ECM) and increased phagocytic activity [34], which one could hypothesize would affect the biodistribution of EVs. However, the current literature is limited and inconsistent. Tong et al. observed clear differences between pregnant and non-pregnant mice in terms of greater accumulation of placental large-EV signals in the lungs of pregnant mice while greater signals were seen in the liver of non-pregnant mice at the same timepoint [34]. In contrast, Tsai et al. did not reproduce this outcome in pregnant mice and showed an approximately 6-fold greater localization of placental large-EVs in the liver than the lungs in pregnant mice [40]. However, unlike Tong et al., Tsai et al. did not compare biodistribution between pregnant and non-pregnant animals within the same experiment.

It is unknown how placental large-EVs are cleared from the body, but, as the majority of large placental EVs seem to be localized to the liver, resident macrophages called Kupffer cells could be responsible for clearing the bulk of the administered EVs [45]. Large-EVs, regardless of their source, are cleared rapidly from the circulation with only ∼30% of the i.v. administered large-EVs remaining in blood after 2 min, reducing to ∼9% at 30 min [45,46], possibly a reflection of the combination of rapid entrapment in tissues and clearance by Kupffer cells and/or other phagocytes. However, a kinetics study is warranted to investigate whether the state of pregnancy, which is characterized by elevated total circulating EVs and cardiovascular changes, could significantly influence the clearance of large-EVs.

### Placental large-EVs and the spleen and kidneys

There is inconsistency in the reported biodistribution of placental large-EVs to the kidneys and the spleen. It is unlikely that EVs in the circulation are cleared via passage into the urine under normal conditions as the glomerular filtration size of ∼5–7 nm in a non-pregnant state poses a significant barrier to the passage of both large- and small-EVs [47,48]. However, Tong et al. reported that placental large-EVs were localized to the kidneys in non-pregnant but not in pregnant mice [34]. We speculate that the putative large-EV signal in the kidneys in non-pregnant mice could be due to the biodistribution of contaminating high-density lipoprotein (HDL) components that have a diameter of 5–10 nm which may pass through the glomerular filtration pore (∼5–7 nm) which have been shown to accumulate in proximal tubule epithelial cells in mouse kidneys [49,50]. During pregnancy, the glomerular pore size is further constricted, especially in late gestation [51], which may challenge this, leading to the evidenced reduction in the accumulation of large-EVs. In contrast with the findings by Tong et al., Tsai et al. reported moderate levels in the kidneys of pregnant mice [40]. The reason behind this inconsistency is currently unknown, although these EVs may have interacted with resident macrophages in the kidney which are known to be phagocytic [52]. Again, studies examining the side-by-side comparison of EV distribution to the kidneys in non-pregnant and pregnant animals are warranted. Furthermore, treating non-pregnant recipient animals with hormones (e.g., progesterone) responsible for regulating the physiological changes to the kidneys during pregnancy would have merit [53].

Contrary to their expectations, Tong et al. did not find placental large-EVs in the spleen and suggested insensitivity in the detection method may have been responsible for this finding. In contrast, Tsai et al. did report localization of placental large-EVs to the spleen using the same imaging system. The inconsistency behind splenic distribution of placental large-EVs between the two studies is again unknown but might be attributed to the different fluorescent dyes that were used in the two studies. Tong et al. employed CellTrace Far Red DDAO-SE that labels EVs luminally [34], whereas, Tsai et al. used near-infrared Cy7 that labels EV surface proteins [40]. Fluorescent dyes that label the surface of EVs, like Cy7, may increase their hydrodynamic size, which can shift their biodistribution towards the spleen [54], a phenomenon that is also seen with larger artificial nanoparticles [55]. Considering the potential influence of EV labels, future EV biodistribution studies should document changes in size following the labelling of EVs. Furthermore, surface labelling techniques may interfere with the interactions between EV surface integrins and their ligands on target cells which may also influence biodistribution of all subtypes of EV [56].

## The biodistribution of placental small-EVs

The six studies investigating placental EV biodistribution have used small-EVs from various species, biofluids and culture models, and are more numerous than the studies examining biodistribution of large-EVs (Table 1).

### Placental small-EVs localize to the lungs

Similar to macro- and large-EVs, the lungs are one of the major sites to which placental small-EVs localize. Following tail vein administration into pregnant and non-pregnant mice, small-EVs derived from human and mouse placental explants showed significant levels of pulmonary localization at all timepoints examined, ranging from 2 min to 24 h [40,57,58]. That the placental small-EV signal in the lungs is retained at a relatively high level for long time periods indicates that the bulk of the placental small-EVs seen in the lungs are specifically targeted to, and taken up by, cells in this organ. In fact, pulmonary distribution of small-EVs and their relatively long retention times is commonly seen in the literature from EVs from diverse sources [46]. However, the specific cell types targeted by EVs depends on the EV donor cell type and is likely driven by the integrins on the EVs. Specific cell types targeted by EVs can be identified by detecting labelled EVs via microscopic visualization of tissue sections and/or flow cytometry of a cell suspension prepared from tissue [56,59,60]. For example, Hoshino et al. demonstrated that integrin α6β1 is involved in the targeting of breast cancer cell-derived small-EVs to fibroblasts and epithelial cells in the lungs in mice [56]. Nguyen et al. demonstrated that plasma-derived small EVs from pregnant mice localized to the lungs and liver whereas, similar EVs from non-pregnant mice did not localize to the lungs [58]. Follow-up experiments demonstrated that murine placental EVs localized specifically to LYVE1+ CD68+ interstitial macrophages, but not alveolar macrophages, and this interaction was mediated by integrins present on the surface of the EVs. An elegant experiment in which transgenic dams expressing mTomato pregnant with female pups that constitutively expressed green fluorescent protein (GFP) demonstrated the passage of GFP-positive placental or fetal EVs to the maternal lungs at gestation day 14.5, appearing as punctate GFP signals in tissue sections [58]. In this experiment, the pups also carried the Cre gene/protein, which was able to excise the mTomato gene from some maternal pulmonary cells suggesting that placental or fetal EV-borne proteins or enzymes can be bioactive *in situ*.

### Placental small-EVs localize to the liver

The liver is another site where large amounts of placental small-EVs accumulate. Multiple studies have shown that following tail vein administration of fluorescently labeled human or mouse placental explant-derived small-EVs, signals were typically concentrated in the liver at all timepoints examined (up to 24 h) [40,57,58]. Tsai et al. reported EV signal intensities in the liver that were ∼7-fold greater than in the lungs or other organs [40]. As the liver is the largest organ in the body and has high blood flow in mice (945 ± 242 ml/min), accumulation of small-EV signals in this organ is not surprising [46]. As the interaction between EVs and endothelial cells, involving rolling, arrest, and accumulation, can take as long as 50 min [61], liver endothelial cell small-EV uptake may be minor, especially early after administration. The pattern of intense biodistribution of placental small-EV signals in the liver at the early timepoint of 30 min, and strong signals remaining at 24 h, is in agreement with the literature describing the biodistribution of small-EVs regardless of their source [46]. Microscopic examination of liver sections has confirmed co-localization of placental small-EVs with Kupffer cells [58]. It has been shown that the capacity of Kupffer cells to take up small EVs is saturated within 90 s of small-EV administration [62]. By blocking placental small-EV surface integrins with an arginine-glycine-aspartate (RGD) peptide, Nguyen et al. deduced that integrins α5β1/αVβ3 were involved in small-placental EV targeting to the liver [58], most likely to fibronectin, a component of the hepatic extracellular matrix [63]. The high blood flow to the liver and the targeting to fibronectin, which is rich in this organ, likely allows a favorable environment for Kupffer cells to easily come into contact with EVs and to facilitate their rapid uptake. The recognition by Kupffer cells may involve a negatively charged phosphatidylserine (PS) that is enriched on small-EV surfaces [64,65], and combinations of integrins on EV surfaces may also facilitate PS-independent uptake [56]. There is a consensus from all EV biodistribution studies that approximately 90% of small-EVs are taken up by Kupffer cells in the liver [46]. In regard to function, while it is possible that small-EVs may deliver specific signals to the liver it seems more likely that uptake by Kupffer cells results in degradation, or clearance, of small-EVs as part of the reticuloendothelial system [46]. However, it should be noted that the liver is a major site in which immunologic tolerance is induced and many of the Kupffer cells function as M2 macrophages and tend to produce cytokines and other factors that leads to tolerance rather than immune activation (reviewed in [66]). It may be a possibility that Kupffer cells that have taken up placental EVs may express fetal minor histocompatibility antigens (derived from the placental EV) that are recognized by various T cells which could contribute in maternal immune tolerance to the fetus.

### Placental small-EVs and the spleen

There is inconsistency in the reported biodistribution of placental small-EVs to the spleen. Tong et al. and Nguyen et al. reported that small-EVs derived from human or mouse placental explants failed to show splenic distribution at all examined timepoints, up to 24 h, following tail vein administration into pregnant or non-pregnant mice [57,58], while Tsai et al. reported splenic distribution of human placental explant-derived small-EVs in pregnant mice [40]. However, the latter study did not perform cardiac puncture to flush the mouse of residual blood prior to organ harvesting, which can lead to significantly exaggerated EV signals in the spleen, indicating that EVs are not readily taken up and retained by splenic cells [67]. Considering this, the spleen does not appear to be a major site to which placental small-EVs localize. This was unexpected as the spleen is an organ responsible for mediating innate and adaptive immune functions with potential involvement in maternal tolerance toward fetal antigens [68]. Furthermore, there is evidence indicating that placental EVs interact with various immune cells *in vitro* [69–72]. Despite this, human placental explant-derived small-EVs have been detected at the microscopic level in spleen tissue sections (our unpublished results) which highlights the limitation of analysing fluorescence at the bulk organ level when biodistribution that can only be detected at the cellular level occurs. This may indicate that a small subset of placental small-EVs can target splenic cells, or a significant portion may possess surface moieties that actively allow escape from splenic cells. Whether these moieties are specific to placental EVs remains to be elucidated, although this seems unlikely given that small-EVs from diverse sources also fail to show splenic distribution [73–76]. Whether this apparently low level of interaction between placental small-EVs and splenic cells is sufficient to create a physiological functional response, such as participation in maternal tolerance, is unclear and warrants investigation.

### Placental small-EVs and the kidneys

The literature is inconsistent regarding the biodistribution of placental small-EVs to the kidneys. While one study reported fluorescently labelled human placental explant-derived small-EV signals could be detected in the kidneys of pregnant mice following tail vein administration with increasing load until 24 hours [57], another failed to show renal distribution of human or mouse placental explant-derived small-EVs at 24 hours in non-pregnant or pregnant mice [58]. As addressed earlier, the glomerular pore size is a barrier against large particles, including small-EVs entering the urine, regardless of pregnancy. However, multiple reports show small-EVs from diverse sources are localized to the kidneys, albeit typically at low levels, following i.v. administration into rodents and macaques [60,77–79]. In pathological conditions, such as preeclampsia or gestational diabetes mellitus, the filtration pore may enlarge, allowing EVs to filter through evidenced by placental EVs present in the urine of women with the latter condition [80]. Alternatively, transcellular movement of intact EVs across the glomerular endothelial cells or the movement of EVs from the peritubular capillary network to the proximal convoluted tubule suggests a possible route for limited renal distribution [81]. These processes may partly explain the slowly increasing accumulation of placental small-EV signals in the kidneys across time [57]. Given the growing recognition of EVs as diagnostic tools [82], the knowledge that placental small-EVs can pass through the kidneys into the urine for non-invasive collection provides grounds for further investigation.

**Figure 2:**
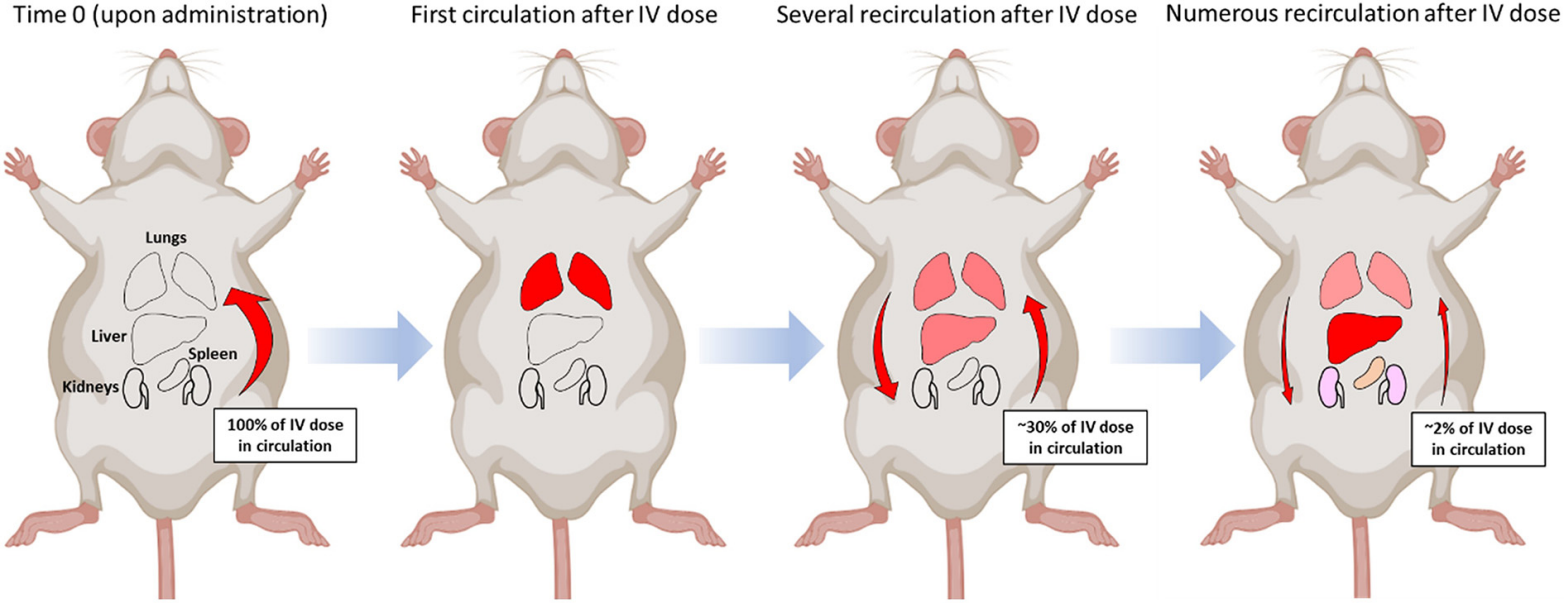
A hypothetical model depicting that the changes in body-wide biodistribution of placental EVs across time following i.v. tail vein administration is dependent on the direction of blood flow, blood recirculation, and retention. Following administration, EVs flood the lungs before disseminating into other organs. Each recirculation delivers EVs into bodily compartments with complementary removal of EVs from the blood. The values of i.v. dose remaining in circulation is taken from a systematic review of EV biodistribution by Kang et al. [46]. Some EVs that are weakly retained in these organs are redistributed to other organs through blood recirculation. Retainment of EVs in organs depends on the cellular uptake of EVs within those organs, which is facilitated by targeting moieties such as integrins and negative charge conferred by phospholipids on the EV surface. Illustrations produced using BioRender and Smart Servier Medical Art, covered by the Created Commons 3.0 license.

Overall, analysis of body-wide biodistribution of placental EVs has been the primary interest in most of the EV biodistribution studies. The biodistribution patterns of placental EVs in maternal organs changed with time, which was dependent on the EV particle size (macro-, large-, and small-EVs), the direction of blood flow, the recirculation of blood which facilitated the gradual redistribution of injected EVs from one organ to another, as well as the retention capacity in organs and tissues (illustrated in Figure 2). While whole animal imaging and whole organ/tissue imaging for labelled EV signals is useful for studying the body-wide biodistribution of placental EVs, a major limitation is the inability to detect and identify low levels of cell-specific biodistribution. In this sense, microscopic visualization and flow cytometry has allowed more refined cell-specific analysis of EV-uptake [83].

## Localization of placental EVs in other maternal tissues

Using a variety of transgenic cells and mice that were examined by confocal microscopy, Sheller-Miller et al. demonstrated that placental EVs were present in the maternal plasma and localized to the uterus and cervix [16]. This group had previously shown that injection of plasma-derived late gestation (day 18 of approximately 20) small-EVs (‘exosomes’) induced preterm birth when administered into mice on gestational day 15, with the intraperitoneal (i.p.) injected small-EVs localizing to the female reproductive tract [85]. Another group has shown that small quantities of fluorescently labeled human placental large- and small-EVs may be localized to the maternal heart after tail vein injection although the signal was very low, near background level [34,57]. The same authors have shown minor and inconsistent localization of EVs to one or more placentae in pregnant mice.

## Passage of maternal small-EVs across the placenta to the fetus

Four studies have collectively investigated the bi-directional movement of EVs across the placental barrier (Table 1). While most studies have been concerned with placental/fetal to maternal direction of EV movement, Shi et al. and Sheller-Miller et al. have reported that murine blood-derived small-EVs administered i.v. or i.p. to pregnant mice can reach the cervix, uterus, placenta, fetal membranes, as well as the fetal heart after 48 h, indicating movement of small-EVs across the placental barrier [84,85]. Interestingly, plasma-derived EVs from gestation day 18 mice, but not gestation day 9 mice, could induce preterm labour in these recipient pregnant mice by creating local inflammatory responses in the uterus, cervix, and fetal membranes, indicating the importance of different EV cargo in creating different physiological outcomes in the presence of identical EV biodistribution. Together with the finding that human amniotic epithelial cell-derived small-EVs administered into the amniotic cavity of pregnant mice can travel to the placenta, maternal circulation, and maternal tissues, this indicates that small-EV movement across the placenta is bi-directional, at least in mice [15]. This is also decisively demonstrated using a transgenic mouse model in which placental or fetal-derived EVs expressing tomato red fluorescent protein could be seen distributed to the maternal uterus and cervix, as well as reaching the maternal circulation [16].

## Future directions

Currently, the literature on placental EV biodistribution presents a somewhat convoluted picture. While there are many reasons for this confusion, a major contributor is the enormous differences between methodologies employed by each study, as well as insufficient number of studies, particularly for placental large-EVs. These differences in methodology are reflective of the general literature on EV biodistribution which is a new field and the limitations of these methods of analysis are discussed in Kang et al. [46].

Given that pregnancy is associated with systemic changes to the maternal anatomy and physiology, future studies investigating placental EV or pregnancy-associated EVs may benefit by studying biodistribution in both pregnant and non-pregnant recipient animals to investigate if biodistribution/function is influenced by the large-scale hormonal and physiological changes during pregnancy [86].

Outside of the setting of pregnancy, administering different doses of EV altered the intensity and pattern of their biodistribution [87,88]. As such, consideration should be given to selecting the experimental EV dose, ideally including a side-by-side comparison of different ranges of EV dose. Furthermore, as different EV isolation techniques influence EV biodistribution patterns, caution is advised when interpreting biodistribution data collected using a single EV isolation technique [89].

Traditionally, i.v. administration of EVs is the most frequently utilized route for investigating biodistribution of EVs, typically with a single bolus. However, this is far from physiologically relevant and may produce misleading biodistribution patterns [67]. Burns et al. and James-Allan et al. have successfully utilized osmotic pumps (Alzet®) installed into recipient animals to allow sustained infusion of small-EVs across several days [83,90]. Combined with the calculation of the rate of total EV secretion from various organs into plasma in mice per minute (∼18 µg of EVs per minute) [65], it would be possible to closely mimic EV exposure by organs and, therefore, their biodistribution in a more physiological setting. Close attention should be given to the potential change in EV half-life across time given the recent finding that EVs are cleared faster following consecutive dosing. This may imply an acquired immune response specific to the administered EVs although other explanations may be possible [60].

Transgenic mouse models in which the placenta express fluorescent proteins are powerful tools to study the physiological biodistribution of placental EVs. This method benefits in several ways, including the tracking of endogenous placental or fetal EVs, avoids overburdening the animal with large EV doses, and may avoid the potential increase in hydrodynamic EV size associated with the exogenous attachment of surface fluorescent labels. Although Sheller-Miller et al. and Nguyen et al. utilized this model, they only investigated EV localization in a few select organs rather than true biodistribution [16,58]. Furthermore, employing guinea pig models to study placental EV biodistribution may be of great value given that humans and guinea pigs share greater anatomical (e.g. deeply invasive placenta) and hormonal (e.g. progesterone levels) similarities during pregnancy compared to the more commonly used mouse models [91]. These methods, however, cannot be used to study the biodistribution of macro-EVs which are essentially specific to primate pregnancy.

Lastly, since the ultimate purpose of EV biodistribution studies is to identify the fate of EVs, techniques that are more sensitive and can focus on cellular localization such as microscopic or flow cytometric evaluation of EV signal localization will need to be employed in future studies.

## Conclusion

Fetal–maternal cross-talk is crucial in maintaining healthy pregnancy and the exchange of EVs between the fetus and its mother is an increasingly recognised role in this conversation. Knowledge of placental EV biodistribution, and therefore the fate of EVs and their functional cargo, is essential in bridging the accumulated wealth of *in vitro* placental EV function to a physiological context. This review has highlighted that the body-wide biodistribution of placental EVs has been the primary interest in past studies but given the lack of knowledge on the quantity of EVs required to manifest an intended response, such as the potential maternal tolerance to fetal antigen in the spleen, minor levels of biodistribution to specific tissues or cells may be equally important. Future consideration should be given to utilizing improved animal models scoping a thorough collection of organs and tissues at both the macroscopic and microscopic level for a fuller understanding of placental EV biodistribution.

## Data Availability

Data openly available in a public repository that issues datasets with DOIs.

## Open Access

Open access for this article was enabled by the participation of The University of Auckland in an all-inclusive *Read & Publish* agreement with Portland Press and the Biochemical Society under a transformative agreement with CAUL.

## Abbreviations

ECM: extracellular matrix
EGFP: enhanced green fluorescent protein
EV: extracellular vesicle
GFP: green fluorescent protein
HDL: high-density lipoprotein
IgG: immunoglobulin G antibody
i.p.: intraperitoneal
i.v.: intravenous
MVB: multivesicular bodies
PLAP: placental-type alkaline phosphate
PS: phosphatidylserine
RGD: arginine-glycine-aspartate
RFP: red fluorescent protein
SNA: syncytial nuclear aggregate
